# Protocol: a rapid and economical procedure for purification of plasmid or plant DNA with diverse applications in plant biology

**DOI:** 10.1186/1746-4811-6-1

**Published:** 2010-01-14

**Authors:** Jian-Feng Li, Li Li, Jen Sheen

**Affiliations:** 1 Department of Genetics, Harvard Medical School, Boston, Massachusetts, USA; 2 Department of Molecular Biology, Massachusetts General Hospital, Boston, Massachusetts 02114-2790, USA

## Abstract

Research in plant molecular biology involves DNA purification on a daily basis. Although different commercial kits enable convenient extraction of high-quality DNA from *E. coli *cells, PCR and agarose gel samples as well as plant tissues, each kit is designed for a particular type of DNA extraction work, and the cost of purchasing these kits over a long run can be considerable. Furthermore, a simple method for the isolation of binary plasmid from *Agrobacterium tumefaciens *cells with satisfactory yield is lacking. Here we describe an easy protocol using homemade silicon dioxide matrix and seven simple solutions for DNA extraction from *E. coli *and *A. tumefaciens *cells, PCR and restriction digests, agarose gel slices, and plant tissues. Compared with the commercial kits, this protocol allows rapid DNA purification from diverse sources with comparable yield and purity at negligible cost. Following this protocol, we have demonstrated: (1) DNA fragments as small as a MYC-epitope tag coding sequence can be successfully recovered from an agarose gel slice; (2) Miniprep DNA from *E. coli *can be eluted with as little as 5 μl water, leading to high DNA concentrations (>1 μg/μl) for efficient biolistic bombardment of *Arabidopsis *seedlings, polyethylene glycol (PEG)-mediated *Arabidopsis *protoplast transfection and maize protoplast electroporation; (3) Binary plasmid DNA prepared from *A. tumefaciens *is suitable for verification by restriction analysis without the need for large scale propagation; (4) High-quality genomic DNA is readily isolated from several plant species including *Arabidopsis*, tobacco and maize. Thus, the silicon dioxide matrix-based DNA purification protocol offers an easy, efficient and economical way to extract DNA for various purposes in plant research.

## Introduction

DNA extraction is a routine procedure in most plant laboratories. Molecular cloning involves DNA purification from *E. coli*, from PCR and restriction digestion mixtures, and from agarose gel slices containing DNA fragments. Genomic DNA often needs to be extracted from plant tissues to facilitate subsequent PCR, sequencing or DNA blot analysis. Although these DNA extractions can be accomplished using commercial kits, each kit is only designed for single-purpose DNA extraction and the cost of purchasing multiple kits can represent a significant research cost. Thus, there is a strong need for a simple and inexpensive protocol that could be adapted to the extraction and purification of DNA from diverse sources.

In plant research, transgenic plants are usually generated using an *Agrobacterium tumefaciens*-mediated transformation procedure, where the bacteria carry an engineered binary plasmid harboring the gene of interest for integration into the plant genome. It is important to isolate the binary plasmid from *A. tumefaciens *cells to verify the correct construct prior to plant transformation since the latter is a relatively lengthy process. However, the extraction of the binary plasmid from *A. tumefaciens *is notoriously tricky due to the low plasmid copy number in *Agrobacterium *and the recalcitrance of the bacteria strain to cell lysis [1]. To solve these problems, lysozyme is often added to the cell lysis solution, while the isolated DNA is usually re-transformed into *E. coli *to propagate before subsequent restriction digestion verification [2]. A simple and reliable protocol for *Agrobacterium *plasmid purification has not been reported.

In an attempt to develop a simple DNA purification method that could meet diverse research needs, we explored the silica-based technique which relies on the ability of DNA to bind to silica particles in the presence of chaotropic salt [3]. By using the cheap chemical compound silicon dioxide as a DNA binding matrix, we have been able to develop individual DNA purification sub-protocols for plasmid miniprep from *E. coli *or *A. tumefaciens*, extraction of DNA fragments from PCR mixtures, restriction digests or agarose gels, and extraction of genomic DNA from plant tissues. During our trial, we have extensively simplified and streamlined these sub-protocols to optimize time and labor efficiency, as well as minimize the effective chemicals to achieve maximal long-term saving without sacrificing the quality of DNA products.

## Materials and methods

### Bacterial strain

*E. coli *strain TOP10 or MC1061 and *A. tumefaciens *strain GV3101 were used in this study.

### Plant growth

*Arabidopsis *was grown in a cycle of 12 h light/23°C followed by 12 h dark/20°C. Tobacco was grown in a cycle of 16 h light/28°C followed by 8 h dark/24°C. Greening maize seedlings were grown at 25°C.

### Plasmid construction

Standard molecular cloning protocols were followed for PCR and plasmid construction. *AtHXK1 *and *AtWRKY29 *with introns were amplified by PCR from *Arabidopsis *genomic DNA using the following primers: *AtHXK1*, forward: CGAGCTAGCATGGGTAA AGTAGCTGTTGGA and reverse: CGAGGATCCAGAGTCTTCAAGGTAGAGAGA; *AtWRKY29*, forward: CGAGCTAGCATGGACGAAGGAGACCTAGAA and reverse: CGAGGATCCGTAATTCCATAAATACCCACT. The PCR product of *AtWRKY29 *was digested with NheI/BamHI and inserted between the NheI/BamHI sites of pAN-GFP vector to obtain the *35S-WRKY29g-GFP *plasmid. A 40-bp DNA fragment containing the MYC-tag (EQKLISEEDL) coding sequence flanked by BamHI/NotI sites was then inserted between the BglII/NotI sites of the *35S-WRKY29g-GFP *plasmid to achieve the *35S-WRKY29g-GFP-MYC *plasmid.

### Transient gene expression and microscopic examination

Particle bombardment and fluorescent imaging were carried out according to the procedure described previously [4]. *Arabidopsis *and maize protoplast transfection was performed as described earlier [5,6].

### Protocol

**Figure 1:**
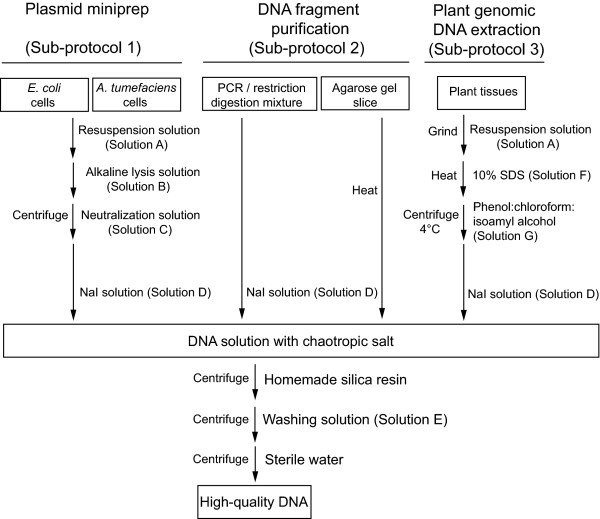
**Overview flowchart of the silica protocol for multiple purpose DNA purification in plant research**. All the procedures were carried out at room temperature unless otherwise indicated. The heating treatment was conducted at 70°C.

All the procedures were carried out at room temperature unless otherwise stated. The use of different solutions is summarized in Figure 1 and their recipes are listed in Table 1.

**Table 1:** Seven solutions used in the silica protocol

Solution Name	Solution Composition	Storage	Usage
Solution A (Resuspension solution)	50 mM Tris-HCl, pH 7.5, 10 mM EDTA, 100 g/ml RNase A	4°C	Plasmid miniprep, genomic DNA extraction

Solution B (Alkaline lysis solution)	0.2 M NaOH, 1% sodium dodecyl sulfate (SDS)	Room temp.	Plasmid miniprep

Solution C (Neutralization solution)	1.32 M KOAc, pH 4.8	Room temp.	Plasmid miniprep

Solution D (NaI solution)	6 M NaI	4°C	Plasmid miniprep, PCR/Gel purification, genomic DNA extraction

Solution E (Washing solution)	50% ethanol, 10 mM Tris-HCl, pH 7.5, 100 mM NaCl, 1 mM EDTA	Room temp.	Plasmid miniprep, PCR/Gel purification, genomic DNA extraction

Solution F	10% SDS	Room temp.	Genomic DNA extraction

Solution G	Phenol:chloroform:isoamyl alcohol (25:24:1, v/v)	4°C	Genomic DNA extraction

Solution D is stored in darkness; Solution G is covered by water phase with β-mercaptoethanol

### Preparation of silica matrix

The procedure to prepare the size-fractionated silica particles was modified from the previous study [7]. Briefly, 5 g silicon dioxide (Sigma, S5631) was mixed with 50 ml sterile water in a 50 ml Falcon tube and settled for 2 h. The supernatant, containing fine silica particles with sizes below ca. 1 μm, was removed and the pellet was resuspended in 50 ml sterile water and re-settled for another 2 h. After discarding the supernatant, the packed silica was resuspended in 50 ml sterile water to make a final concentration of approximately 100 mg/ml. One mg of the silicon dioxide is able to bind 3-4.5 μg DNA [7]. The slurry can be stored at room temperature and will be stable for at least 12 months.

### Sub-protocol 1 (For plasmid miniprep from *E. coli *or *A. tumefaciens*)

1. Pellet the cells of 2 ml *E. coli *overnight culture (OD600 = 2.0) growing at 37°C or *A. tumefaciens *overnight culture (OD600 = 2.0) growing at 28°C by centrifugation at 16,000 g for 30 sec.

2. Resuspend the cell pellet in 100 μl Resuspension solution (Solution A) by brief vortexing.

3. Add 100 μl Alkaline lysis solution (Solution B) and invert the microfuge tube for several times.

4. Let the tube sit for 2 min for *E. coli *or 5 min for *A. tumefaciens *at room temperature.

5. Add 100 μl Neutralization solution (Solution C) and invert the tube for a few times.

6. Centrifuge at 16,000 g for 5 min and transfer the supernatant to a fresh tube containing 500 μl 6 M NaI solution (Solution D) and mix well by inverting the tube.

7. Add 20 μl silica matrix, mix well and let the tube sit at room temperature for 2 min.

NOTE: *Extending the incubation time to 5 min could slightly increase the yield*.

8. Pellet the matrix by centrifugation for 10 sec at 16,000 g. Pour off the supernatant and gently tap the inverted tube against a Kimwipe to drain the liquid.

9. Wash the matrix by resuspending with 500 μl Washing solution (Solution E) and vigorously vortexing.

10. Repeat step 8 and 9.

11. Pellet the matrix by centrifugation for 10 sec at 16,000 g and remove the supernatant by pipetting.

12. Centrifuge for another 10 sec and carefully pipette off the residual liquid.

13. Add 40 μl sterile water to resuspend the pellet by brief vortexing and place the microfuge tube at 70°C for 2 min.

14. Centrifuge at 16,000 g for 2 min and transfer 36-38 μl supernatant containing the eluted plasmid DNA to a fresh tube.

NOTE: *The remaining 2-4 μl liquid should be abandoned due to a slight contamination by disturbed silica particles. For protection against DNase digestion or pH fluctuations, TE buffer (10 mM Tris-Cl, pH8.0, 1 mM EDTA) should be used to elute DNA*.

### Sub-protocol 2 (For DNA purification from solution or agarose gel slice)

1. Add 150 μl 6 M NaI (Solution D) to up to 50 μl PCR or restriction digestion mixture and mix well by inverting the tube. In case of the agarose gel slice containing DNA fragments, add 300 μl Solution D for every 100 mg gel slice and heat the microfuge tube at 70°C for 3 min to dissolve the gel.

2. Add 10 μl silica matrix to the blend, mix well and incubate for 2 min at room temperature.

NOTE: *Extending the incubation time to 5 min could slightly increase DNA recovery rate*.

3. Pellet the matrix by centrifugation for 10 sec at 16,000 g and remove the supernatant by pipetting.

4. Wash the matrix in 500 μl Washing solution (Solution E) by vigorously vortexing.

5. Repeat step 3 and 4.

6. Pellet the matrix by centrifugation for 10 sec at 16,000 g and discard the supernatant.

7. Centrifuge for another 10 sec and pipette off the trace amount of liquid.

8. Resuspend the matrix in 5-30 μl sterile water and place the microfuge tube at 70°C for 2 min.

9. Centrifuge at 16,000 g for 2 min and transfer the DNA eluate into a fresh tube.

NOTE: *For protection against DNase digestion or pH fluctuations, TE buffer (10 mM Tris-Cl, pH8.0, 1 mM EDTA) should be used to elute DNA*.

### Sub-protocol 3 (For plant genomic DNA extraction)

1. Place approximately 10 mg of plant material in a 1.5 ml microfuge tube.

2. Add 200 μl Resuspension solution (Solution A) and grind the tissue using a Micro-Grinder homogenizer (Research Products International Corporation).

3. Add 30 μl 10% SDS (Solution F) to the homogenate and invert the tube for several times.

4. Incubate the tube at 70°C for 10 min.

5. Add 250 μl Phenol:chloroform:isoamyl alcohol (25:24:1, v/v; Solution G) and vortex the mixture vigorously for 30 sec.

6. Centrifuge at 4°C at 16,000 g for 5 min and transfer the aqueous (upper) phase to a fresh tube containing 500 μl 6 M NaI (Solution D) and mix well by inverting the tube.

7. Add 20 μl silica matrix and let the tube sit at room temperature for 2 min.

8. Pellet the matrix by centrifugation at 16,000 g for 10 sec and remove the supernatant by pipetting.

9. Wash the matrix by resuspending with 1 ml Washing solution (Solution E) and vigorously vortexing.

10. Repeat step 8 and 9.

11. Pellet the matrix by centrifugation at 16,000 g for 10 sec and remove the supernatant using a pipette.

12. Centrifuge for another 10 sec and carefully pipette off the residual liquid.

13. Add 40 μl sterile water to resuspend the pellet by brief vortexing and place the microfuge tube at 70°C for 2 min.

14. Centrifuge at 16,000 g for 2 min and transfer 36-38 μl supernatant containing the eluted genomic DNA to a fresh tube.

NOTE: *The remaining 2-4 μl liquid should be abandoned due to a slight contamination by disturbed silica particles. For protection against DNase digestion or pH fluctuations, TE buffer (10 mM Tris-Cl, pH8.0, 1 mM EDTA) should be used to elute DNA*.

## Comments

### Plant genomic DNA purification by the silicon dioxide matrix

**Figure 2:**
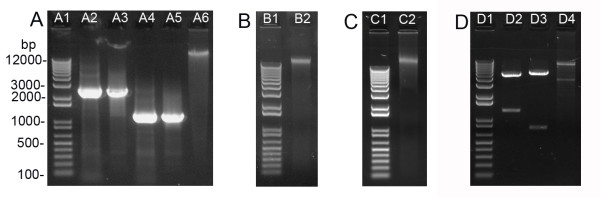
**High quality DNA purified by the silica protocol**. Gel electrophoresis pictures of PCR products, plant genomic DNA and miniprep DNA are shown. A, Genomic PCR products of *AtHXK1 *and *AtWRKY29 *gene and *Arabidopsis *genomic DNA purified by silica matrix. The PCR products of *AtHXK1 *(A2 and A3) and *AtWRKY29 *(A4 and A5) were split into two aliquots with equal amount of DNA. One aliquot was directly subject to electrophoresis (A2 and A4) while the other was purified by silica matrix before electrophoresis (A3 and A5). Genomic DNA (500 ng) purified from 4-week-old *Arabidopsis *leaves by silica matrix was loaded in A6. B, Genomic DNA purified from 7-day-old tobacco seedlings by silica matrix. C, Genomic DNA purified from 10-day-old maize leaves by silica matrix. D, Restriction digestion verification of plasmids minipreped from *E. coli *and *A. tumefaciens *cells following the silica protocol. The *35S-WRKY29g-GFP-MYC *plasmid (400 ng) minipreped from *E. coli *was digested by NheI/BamHI (D2) or BamHI/NotI (D3) before electrophoresis. The binary plasmid *VKH-NLS-YFP-GUS *(200 ng, [12]) prepared from *A. tumefaciens *was digested by SacI/HindIII (D4) before electrophoresis. A1, B1, C1 and D1 correspond to 1 kb DNA ladder with size of each fragment indicated.

Following Sub-protocol 3, high quality genomic DNA could be obtained from 7-day-old *Arabidopsis *seedlings (Table 2), 4-week-old *Arabidopsis *leaves (Figure 2A and Table 2), 7-day-old *Nicotiana benthamiana *seedlings (Figure 2B and Table 2) and 10-day-old maize leaves (Figure 2C and Table 2). The entire procedure from grinding tissue to eluting genomic DNA took around 35 min. Older plant tissues accumulate secondary metabolites and polysaccharides, which often contaminate the extracted genomic DNA due to co-precipitation with nucleic acids during ethanol/isopropanol precipitation [8]. Since only nucleic acids selectively bind to silicon dioxide, they are less likely to be a problem during silica-mediated DNA purification. On the other hand, RNA contamination is completely removed by RNase A included in Resuspension solution (Solution A; Figure 2A, lane A6, Figure 2B and 2C). The *Arabidopsis *genomic DNA prepared in this way could be immediately used as PCR template (Figure 2A).

**Table 2:** DNA yield or recovery rate during silica-mediated purification in this study

Material	DNA	Sub-protocol	Input	DNA production^a^	
				Yield	OD260/280
*E. coli*	*35S-WRKY29g-GFP-MYC*	1	2 ml culture OD600 = 2	6.2 ± 0.3 μg	1.91 ± 0.03
*E. coli*	pCB302	1	2 ml culture OD600 = 2	2.6 ± 0.4 μg	1.93 ± 0.03
*E. coli*	pBI101	1	2 ml culture OD600 = 2	2.4 ± 0.3 μg	1.92 ± 0.02
*E. coli*	pPZP222	1	2 ml culture OD600 = 2	4.7 ± 0.4 μg	1.88 ± 0.02
*A. tumefaciens*	*VKH-NLS-YFP-GUS*	1	2 ml culture OD600 = 2	0.4 ± 0.03 μg	1.95 ± 0.03
DNA solution	*AtWRKY29 *gPCR	2	1 μg DNA	78 ± 6%^b^	1.81 ± 0.05
Agarose gel	*AtWRKY29 *gPCR	2	0.5 μg DNA	68 ± 2%^b^	1.85 ± 0.02
*Arabidopsis *(s)^c^	genomic DNA	3	10 mg tissue	1.7 ± 0.1 μg	1.90 ± 0.02
*Arabidopsis *(l)^d^	genomic DNA	3	10 mg tissue	1.1 ± 0.1 μg	1.93 ± 0.02
tobacco (s)^e^	genomic DNA	3	10 mg tissue	1.9 ± 0.2 μg	1.93 ± 0.01
maize (l)^f^	genomic DNA	3	10 mg tissue	1.0 ± 0.1 μg	1.88 ± 0.03

a. Qualities and quantities of DNA products were measured by NanoDrop ND-1000 Spectrophotometer (Thermo Scientific) and each DNA purification was repeated for at least 5 times.

b. DNA recovery rate (i.e., yield/input × 100%) is shown instead of DNA yield.

c. Severn-day-old *Arabidopsis *seedlings (s) were used

d. Four-week-old *Arabidopsis *leaves (l) were used

e. Seven-day-old tobacco seedlings (s) were used

f. Ten-day-old maize leaves (l) were used

### PCR and gel purification by the silicon dioxide matrix

DNA in PCR mixtures or restriction digests could be easily purified by silica matrix according to Sub-protocol 2. The DNA recovery rate was estimated to be 70-80% depending on the size of the DNA fragment (Figure 2A, lane A2-5; Table 2). This procedure took about 8 min. We have also tried to purify PCR or restriction digestion products from agarose gel slice. In this case, a slightly reduced DNA recovery rate (i.e., 68%, Table 2) was obtained and an extra heating step was required to dissolve the agarose gel in NaI solution (Solution D), which would take two more minutes when compared with a direct DNA purification from solution.

**Figure 3:**
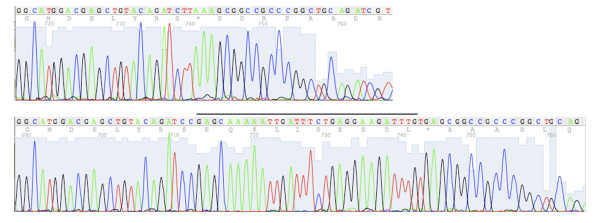
**Miniprep DNA by the silica protocol allows a long read length in DNA sequencing**. The 3' end of each DNA sequencing result beyond 700 bp is shown with individual nucleotide peaks clearly distinguishable. The alignment of the sequencing results of *35S-WRKY29g-GFP *plasmid (upper panel) and its derivative *35S-WRKY29g-GFP-MYC *(lower panel) validated a successful insertion of a MYC-epitope tag coding sequence between the BglII/NotI sites in the *35S-WRKY29g-GFP *plasmid. The MYC-tag (EQKLISEEDL) coding sequence is labeled by a line on top. The numbers underneath the DNA sequence were generated by the sequence reading software 4Peaks http://mekentosj.com/science/4peaks/ to indicate the read length. Note that the BglII site of the *35S-WRKY29g-GFP *plasmid was removed after ligation to the BamHI site in front of the MYC-tag coding sequence.

Purification of small (< 50 bp) DNA fragments is rather challenging even when the commercial DNA purification kit is used. This is because short DNA tends to bind tightly to the column matrix and is difficult to elute, and is also difficult to precipitate during ethanol/isopropanol precipitation. To test whether Sub-protocol 2 is also suitable for purifying small DNA fragments from agarose gel slice, a 40-bp DNA fragment composed of the MYC-epitope tag coding sequence, BamHI and NotI sites was resolved in a 2.5% agarose gel and was purified from the gel slice using silica matrix. The DNA eluate was used in ligation with BglII and NotI double digested *35S-WRKY29g-GFP *plasmid leading to a successful insertion of the short DNA fragment into the plasmid (Figure 3). This result suggested that the silica protocol enabled the purification of short DNA fragments, which presumably benefited from the heating step during DNA elution that greatly facilitated the small DNA elution from the silica matrix.

### Alkaline lysis combined with silica matrix for plasmid miniprep from *E. coli*

Plasmid miniprep from 2 ml *E. coli *cells following Sub-protocol 1 takes approximately 15 min to obtain DNA. The yield of *35S-WRKY29g-GFP-MYC*, a 6 kb high copy number plasmid, was about 6.2 μg (Table 2). To evaluate the miniprep yield of low copy number or larger plasmids by the silica protocol, several binary vectors frequently used in plant transformation were tested. Low-copy binary vectors pCB302 (5 kb, [9]) and pBI101 (12 kb, [10]) as well as high-copy binary vector pPZP222 (8.7 kb, [11]) were minipreped and the yields were 2.6 μg for pCB302, 2.4 μg for pBI101 and 4.7 μg for pPZP222 (Table 2). In addition to high DNA yield, silica-mediated miniprep also offers great DNA quality (Table 2). The plasmid DNA prepared herein could be readily digested by restriction enzymes (Figure 2D) or used as PCR template (data not shown). Importantly, the resultant DNA could generally yield sequence read length beyond 700 bp during DNA sequencing analysis (Figure 3), which also reflected the high quality of the DNA template generated by the silica protocol.

**Figure 4:**
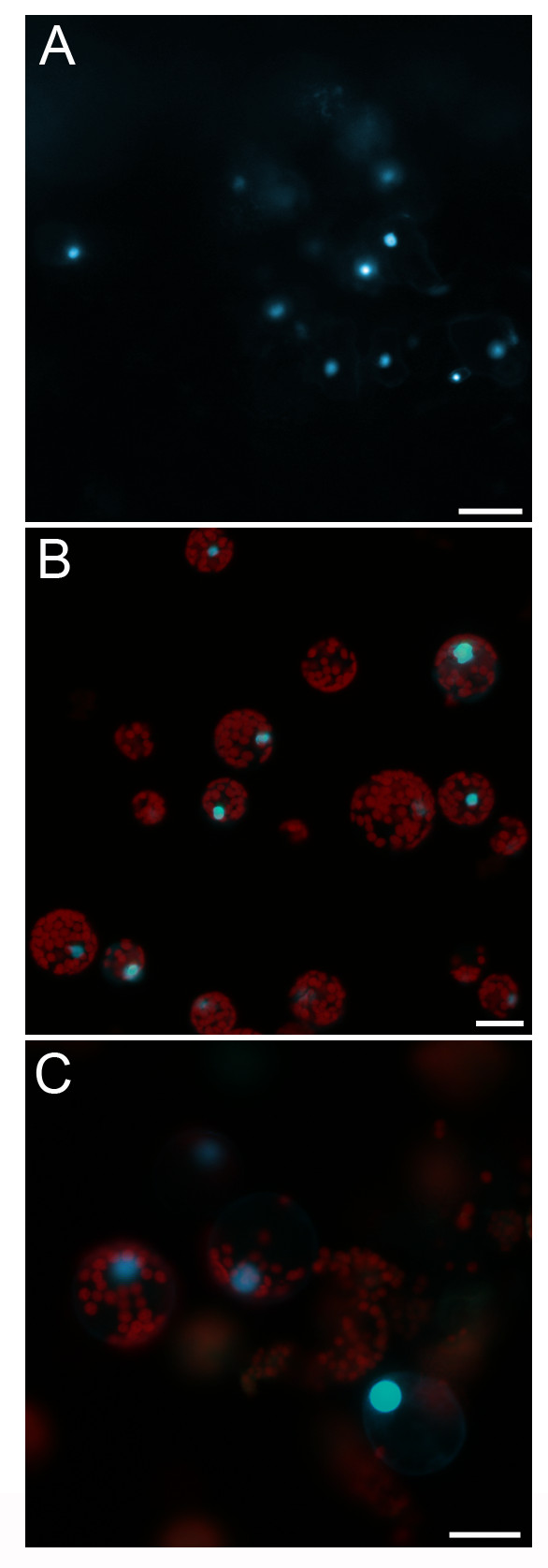
**Miniprep DNA eluted from silica matrix at high concentration allows efficient transient gene expression assays**. A, Cotyledon cells of *Arabidopsis *seedling expressing the WRKY29-GFP-MYC protein in the nucleus. Miniprep DNA (2 μg in 1.8 μl) was bombarded into 7-day-old *Arabidopsis *seedlings and the observation was made 12 h post biolistic bombardment. B, *Arabidopsis *mesophyll protoplasts expressing the WRKY29-GFP-MYC protein in the nucleus. Miniprep DNA (2.2 μg in 2 μl) was introduced into 4,000 *Arabidopsis *mesophyll protoplasts by PEG-mediated transfection and the observation was made 12 h after transfection. C, Maize mesophyll protoplasts expressing the WRKY29-GFP-MYC protein in the nucleus. Fifteen μg of miniprep DNA was used to transfect 10^6 ^maize protoplasts by electroporation and the observation was made 12 h after transfection. Scale bar = 20 μm.

We assessed whether plasmid DNA prepared by the silica protocol could be directly used in transient gene expression assays such as particle bombardment [4] and protoplast transfection [5,6]. The key for efficient transient gene expression is high purity DNA at high concentration, which is particularly important for successful PEG-mediated *Arabidopsis *protoplast transfection [5]. Taking advantage of the heating step during DNA elution, we were able to effectively elute plasmid DNA from silica matrix using only a small volume (e.g., 5 μl) of water thus could maximize the DNA concentration in the eluate. We randomly selected 10 single colonies harboring the *35S-WRKY29g-GFP-MYC *plasmid for miniprep. The DNA concentration in the 5 μl eluate was ranging from 1.1 to 1.4 μg/μl. It is more difficult to obain the same concentrated DNA eluate when the commercial miniprep kit is used. To evaluate the low DNA transfection limit, we chose the miniprep DNA with the lowest concentration (i.e., 1.1 μg/μl) for both types of transient expression assays. Two μg (1.8 μl) of this miniprep DNA was delivered into 7-day-old *Arabidopsis *seedlings by particle bombardment. Hundreds of cells in the cotyledons were found to express the WRKY29-GFP-MYC protein in the nucleus 12 h post bombardment (Figure 4A). This transformation efficiency is roughly the same as that obained by using equal amount of kit-minipreped DNA in bombardment (data not shown). This silica-minipreped DNA (2.2 μg in 2 μl) was also used to transfect 4,000 *Arabidopsis *mesophyll protoplasts and the expression of the WRKY29-GFP-MYC protein was detected in the nucleus of approximately 70% of the protoplasts 12 h after PEG-mediated transfection (Figure 4B). This transfection efficiency is comparable to that achieved by using CsCl gradient maxipreped DNA in protoplast transfection [5]. In addition, we also tested the miniprep DNA in transient gene expression using maize mesophyll protoplasts. In this case, plasmid DNA was introduced into maize protoplasts by electroporation, where low quality DNA containing high salt or other impurities would lead to the failure of electroporation [6]. Successful expression of the WRKY29-GFP-MYC protein was observed in the nucleus of numerous maize protoplasts 12 h after 15 μg of miniprep DNA was used to transfect 10^6 ^maize protoplasts by electroporation (Figure 4C). These data suggested that the miniprep DNA purified by the silica protocol could be readily used for efficient transient assays with no need for further purification or concentration.

### The silica method for plasmid miniprep from *A. tumefaciens*

Plasmid miniprep from 2 ml *A. tumefaciens *cells containing high-copy number binary plasmid *VKH-NLS-YFP-GUS *[12] was conducted according to Sub-protocol 1. After Alkaline lysis solution (Solution B) was added to the cell resuspension, we lengthened the incubation period to 5 min to improve the cell lysis since *Agrobacterium *was more resistant to alkaline lysis than *E. coli*. The DNA yield from *A. tumefaciens *cells by this protocol was about 400 ng (Table 2), which was sufficient for restriction digestion analysis (Figure 2D, lane D4). Binary plasmids with different sizes or copy numbers had similar yields when minipreped from *A. tumefaciens *cells (data not shown). Thus, a quick confirmation of the correct construct in *Agrobacterium *by restriction digestion is feasible using the silica-prepared binary plasmids. The routine re-transformation of the DNA into *E. coli *for propagation [2] is unnecessary.

### Advantages of the silicon dioxide matrix protocol

The silica matrix-based protocol described here offers two major advantages over other DNA purification methods. The first advantage is its low cost. Silicon dioxide itself is a very cheap chemical. The cost of silica matrix consumed in each DNA miniprep is less than one tenth of a U.S. cent and thus is fairly affordable. All solutions used in this protocol are made from common and inexpensive chemicals except NaI (Sigma, 217638). Even after accommodating the expense of NaI, the total costs for each miniprep and each PCR/gel purification are 0.15 and 0.05 U.S. dollar, respectively. A second advantage of the silica protocol is its versatility. Instead of purchasing different commercial kits to meet different DNA purification needs, one could utilize the same DNA binding matrix together with Solution A-G (Table 1) for all the DNA purification work in plant research. Besides silicon dioxide, only Solutions D and E are used by all the DNA extraction applications. The former facilitates DNA binding to silica matrix and the latter washes away nonspecifically bound impurities. Solution A is shared by plasmid miniprep and plant genomic DNA extraction, where the inclusion of RNase A removes RNA contamination from DNA products.

In addition, there are a few minor advantages in using silicon dioxide matrix instead of the column, the latter being used in most of the commercial DNA purification kits. First, the amount of silicon dioxide matrix used in each DNA purification could be flexible depending on the anticipated DNA amount in the material. Second, the silica matrix could be readily separated from the supernatant by a quick centrifugation in less than 10 sec. Third, avoiding liquid retention by the column matrix, DNA binding to the silicon dioxide matrix could be efficiently eluted with a very small volume (e.g., 5 μl) of water. Fourth, the matrix resuspension could be easily heated to facilitate DNA elution, whereas the column is inconvenient for heating treatment. Therefore, this silica protocol not only allows scarce and precious DNA fragments to be effectively recovered in a small volume of water thus maintaining an optimal concentration for DNA ligation, but also allows the miniprep DNA to be eluted from the silicon dioxide matrix in sufficiently high concentrations for efficient protoplast transfection.

## Conclusion

The silicon dioxide matrix-based DNA purification protocol presented here allows fast, simple and economical purification of high quality DNA for multiple purposes in plant research. No specialized device such as column or vacuum apparatus and no additional time-consuming and yield-reducing precipitation steps are required. In principle, we envisage that the DNA preparation by the silica protocol could be easily scaled up to generate a large amount of pure DNA and is promising for high-thoughput DNA purification applications.

## Competing interests

The authors declare that they have no competing interests.

## Authors' contributions

JFL designed the experiments. JFL and LL performed the experiments. JFL and JS wrote the manuscript. All authors read and approved the final manuscript.
